# Herbal medicine for post-stroke insomnia: A protocol for systematic review and meta-analysis

**DOI:** 10.1097/MD.0000000000026223

**Published:** 2021-06-04

**Authors:** Sang-Ho Kim, Jung-Hwa Lim

**Affiliations:** a Department of Neuropsychiatry of Korean Medicine, Pohang Korean Medicine Hospital, Daegu Haany University, 411 Saecheonnyeon-daero, Nam-gu, Pohang-si, Gyeongsangbuk-do; b Department of Neuropsychiatry, School of Korean Medicine, Pusan National University, 49 Busandaehak-ro, Mulgeum-eup, Yangsan, Republic of Korea.

**Keywords:** herbal medicine, insomnia, protocol, stroke, systematic review

## Abstract

**Background:**

Post-stroke insomnia (PSI) is a highly prevalent complication in patients with stroke. However, there has been no comprehensive systematic review assessing the efficacy and safety of herbal medicine (HM) on PSI. This protocol was developed to conduct a systematic review and meta-analysis to evaluate the evidence related to the efficacy and safety of HM on PSI.

**Methods:**

We will perform a comprehensive electronic search, including Medline, EMBASE, CENTRAL, AMED, CINAHL, PsycARTICLES, and Chinese, Korean, and Japanese databases from their inception to November 2020. This systemic review will include only randomized controlled clinical trials of HM on PSI. The main outcome is the Pittsburgh Sleep Quality Index score. Two researchers will independently screen citations and abstracts, identify full-text articles for inclusion, extract data, and appraise the quality and risk of bias of included studies. A meta-analysis will be conducted using Review Manager 5.4. The evidence quality of each outcome will be appraised according to Grades of Recommendation, Assessment, Development, and Evaluation.

**Results:**

This protocol adheres to the Preferred Reporting Items for Systematic Reviews and Meta-Analyses-P (PRISMA-P) guidelines to ensure clarity and completeness of reporting in all phases of the systematic review.

**Conclusion:**

This study will provide evidence regarding the efficacy and safety of HM for the treatment of PSI.

**Ethics and dissemination:**

No ethical approval will be needed because data from previously published studies in which informed consent was obtained by primary investigators will be retrieved and analyzed. We will publish this systematic review in a peer-reviewed journal.

**OSF registration DOI:**

10.17605/OSF.IO/PEHQZ.

## 1 Introduction

Stroke, the second leading cause of death and disability worldwide, is responsible for approximately 11% of total mortality, according to the World Health Organization's 2019 Global Health Estimates.^[1]^ Stroke continues to increase the global burden of disease and exert a heavy pressure on affected individuals, their families, and society.^[2]^ Stroke survivors commonly suffer from long-term sequelae, including impairments in speech and language, swallowing, memory, and visuospatial and perceptual skills; emotional problems; difficulties with daily activities; and physical disabilities.^[3–6]^ Stroke also increases the risk of several secondary diseases, including dementia, fracture, myocardial infarction, cardiac arrhythmias, and cardiac arrest,^[7–10]^ but especially mental health problems, such as depression, anxiety, and insomnia.^[11–13]^

Insomnia is defined as difficulty initiating or maintaining sleep, or early morning awakening for at least 3 nights per week over a period of at least 3 months that affects daytime functioning, according to standard diagnostic criteria.^[14]^ Post-stroke insomnia (PSI) is a highly prevalent complication in patients with stroke. A recent meta-analysis revealed that PSI is common; using the diagnostic tools for insomnia, approximately 32.21% of patients with stroke are affected, which increases to 40.70% when considering insomnia symptoms only.^[13]^ Moreover, there is evidence of a 2-way relationship between stroke and sleep.^[15]^ Insomnia is associated with higher mortality and significantly lower quality of life (QoL) in patients with stroke^[16,17]^ and poor sleep is a risk factor for stroke and worsens stroke outcomes.^[18,19]^ In previous studies, the stroke group with chronic insomnia was more likely to be depressed, anxious, and disabled than the stroke group without chronic insomnia, and more insomnia symptoms were associated with comorbid depression and anxiety.^[13,20]^ Ischemic stroke disrupts sleep architecture and the endogenous circadian rhythm, which indicates that stroke and sleep are bidirectionally related.^[21]^ As insomnia is a potentially modifiable risk factor, it is important to address PSI to improve stroke outcomes.

Pharmacotherapy is commonly used for the management of PSI. Two studies have shown that zolpidem, the most widely used hypnotic drug, might exert neuroprotection and improve stroke recovery.^[22,23]^ However, it neutralizes sleep-dependent neuroplasticity in an animal model with a chronic state of stroke^[24]^ and even increases the risk for ischemic stroke, especially at higher doses.^[25]^ Benzodiazepines, also common hypnotic drugs, are associated with various side effects, dependency, and rebound symptoms after discontinuation.^[26]^ Although cognitive behavioral therapy for insomnia is a first-line nonpharmacological treatment, it is not effective or available for some patients.^[27]^ Therefore, effective and safe alternative medications are needed to treat PSI.

Herbal medicine (HM) has been widely used in the treatment of diseases for thousands of years in East Asia. As it exerts effects through multi-component, multi-target, and multi-pathway mechanisms, HM has been proposed as an alternative to conventional pharmacology.^[28]^ Recent systematic reviews have shown that HM can significantly relieve insomnia.^[29–33]^ However, there is only one retrospective study on the treatment of PSI using HM plus alprazolam.^[34]^

There is only one Chinese systematic review of HM for the treatment of PSI.^[35]^ However, as this previous review is out-of-date, not comprehensive, and unregistered, an updated and comprehensive synthesis of relevant studies is needed to propose optimal recommendations based on the efficacy and safety of HM in the treatment of PSI. Therefore, we will perform an updated and expanded meta-analysis to provide reliable evidence regarding the efficacy and safety of HM in the treatment of PSI.

## 2 Materials and methods

### 2.1 Study registration

We will report this review in accordance with the Preferred Reporting Items for Systematic Reviews and Meta-Analyses (PRISMA) guidelines.^[36]^ The protocol for this review has been registered in the open science framework (OSF) under registration DOI of 10.17605/OSF.IO/PEHQZ.

### 2.2 Inclusion and exclusion criteria

#### 2.2.1 Types of studies

We will include only randomized controlled trials (RCTs) of HM in the treatment of PSI in this review, regardless of publication or language restriction. If the term “randomization” is mentioned without a detailed randomization method, it will still be included in this review. Non-RCTs, case series, uncontrolled trials, reviews, and experimental studies, and quasi-RCTs using inappropriate random sequence generation methods such as alternate allocation will be excluded.

#### 2.2.2 Types of participants

We will include patients with a diagnosis of insomnia following a stroke, using standard imaging tools such as brain-computed tomography, brain-magnetic resonance imaging, brain-magnetic resonance angiography, and perfusion imaging. Only studies using standardized diagnostic criteria of insomnia, such as the Diagnostic and Statistical Manual of Mental Disorders, International Classification of Diseases, International Classification of Sleep Disorders, Chinese Classification of Mental Disorders, and Guiding Principles for Clinical Research on New Drugs of Traditional Chinese Medicine will be included. We will exclude patients with other psychiatric problems, such as depression, anxiety, drug allergies, or other serious illnesses, including cancer, liver disease, and kidney disease. Patients included in studies that do not provide diagnostic criteria or validated tools will be excluded. There will be no restrictions on the sex, age, or race of the participants.

#### 2.2.3 Types of intervention

##### 2.2.3.1 Experimental intervention

Studies that involve oral HM as monotherapy with or without routine care for stroke, such as anti-hypertensive medications and rehabilitation, as the experimental intervention will be included. Any formulation of HM prescribed based on traditional East Asian medicine theories will be allowed. Studies that involve oral HM as adjunctive therapy to psychotropic drugs, such as hypnotics, anxiolytics, and antidepressants, and those that involve acupuncture/acupressure, moxibustion, herbal bathing, music therapy, and psychotherapy as experimental adjunctive interventions will be excluded. Studies that do not list the composition of the HM used, unless patented, will be excluded.

##### 2.2.3.2 Control intervention

Studies that involve psychotropic drugs, such as hypnotics, anxiolytics, and antidepressants, with or without routine care for stroke, such as anti-hypertensive medications and rehabilitation, as control interventions will be included.

#### 2.2.4 Types of outcome measures

The primary outcome will be the changes in the degree of insomnia, as measured using validated assessment tools, such as the Pittsburgh Sleep Quality Index. The total effective rate (TER) and number of adverse events will be included as secondary outcome measures. The TER is a non-validated outcome measure that is processed secondarily to certain evaluation criteria, such as the improvement rates of quantified outcomes or clinical symptoms.

### 2.3 Search methods

The following databases will be searched comprehensively from their inception to November 25, 2020: 6 English-language databases (Medline via PubMed, EMBASE via Elsevier, the Cochrane Central Register of Controlled Trials, Allied and Complementary Medicine Database via EBSCO, Cumulative Index to Nursing and Allied Health Literature via EBSCO, and PsycARTICLES via ProQuest), 5 Korean-language databases (Oriental Medicine Advanced Searching Integrated System, Korean Studies Information Service System, Research Information Service System, Korean Medical Database, and Korea Citation Index), 3 Chinese databases (China National Knowledge Infrastructure, Wanfang Data, and VIP Database), and 1 Japanese database (Citation Information by NII). We also will check the reference lists of relevant articles and manually searched Google Scholar to identify any additional gray literature. There will be no restriction on language or publication status. The following search terms will be used in Medline: (“Sleep” [MH] OR “sleep wake disorders” [MH] OR sleep∗ OR insomnia∗ OR wakeful∗ OR sleepless∗ OR dyssomnia∗) AND (“stroke” [MH] OR stroke) AND (“Plants, Medicinal” [MH] OR “Drugs, Chinese Herbal” [MH] OR “Medicine, Chinese Traditional” [MH] OR “Medicine, Kampo” [MH] OR “Medicine, Korean Traditional” [MH] OR “Herbal Medicine” [MH] OR “Prescription Drugs” [MH] OR “traditional Korean medicine” OR “traditional Chinese medicine” OR “traditional Oriental medicine” OR “Kampo medicine” OR “alternative medicine” OR “complementary medicine” OR herb∗ OR decoction∗ OR botanic∗) (Appendix 1, Supplemental Digital Content).

### 2.4 Data collection and analysis

#### 2.4.1 Literature selection

**Figure 1:**
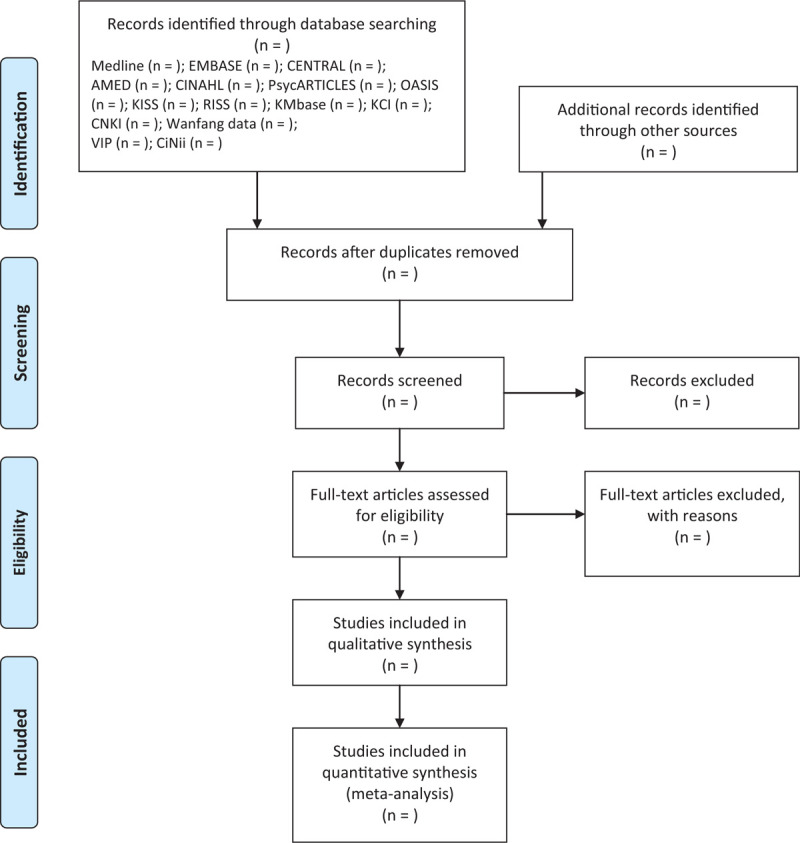
PRISMA flow chart for the study selection process. PRISMA = the Preferred Reporting Items for Systematic Reviews and Meta-Analyses.

All studies retrieved using the search strategy will be imported into EndNote (X9) and duplicated studies will be filtered. The titles and/or abstracts of studies retrieved and those from additional sources will be screened independently by 2 researchers (SHK and JHL) to identify studies that meet the inclusion criteria. The full texts of these potentially eligible studies will then be retrieved and independently assessed for eligibility by same 2 researchers. Any disagreements regarding the eligibility of a particular study will be resolved through discussion. Figure 1 shows the process of literature screening.

#### 2.4.2 Data extraction

A standardized data collection form in Excel 2010 (Microsoft, Redmond, WA) will be used to extract data from the included studies. One author (SHK) will conduct data extraction and another author (JHL) will review the data. Discrepancies will be resolved through discussion. Items extracted from each study will include the first author's name; year of publication; country; sample size and number of dropouts; diagnostic criteria; details about the participants, experimental intervention, and comparisons; duration of the intervention; outcome measures; results; adverse events associated with interventions; and information for the assessment of the risk of bias. Missing data will be requested from the study authors via e-mail if additional information is needed.

### 2.5 Risk of bias assessment

Two researchers (SHK and JHL) will independently assess the methodological quality using the risk of bias tool developed by the Cochrane group.^[37]^ Discrepancies will be resolved through discussion. The risk of bias tool includes selection bias (random sequence generation and allocation concealment), performance bias (blinding of participants and personnel), detection bias (blinding of outcome assessment), attrition bias (completeness of outcome data), reporting bias (selective reporting), and other biases. We will assess the study to be at high risk of bias in the random sequence generation domain when the term “randomization” is mentioned without specific randomization methods. We will assess other potential bias categories, with particular emphasis on baseline imbalances, between experimental and control groups, such as participant characteristics, which include mean age and baseline insomnia level.

### 2.6 Data analysis

#### 2.6.1 Measures of treatment effect

We will pool the data, including mean difference (MD) for continuous outcomes and risk ratio (RR) for binary outcomes, with 95% confidence intervals (CIs).

#### 2.6.2 Assessment of heterogeneity

Heterogeneity between studies will be assessed using both the chi-squared test and *I*^2^ statistic. We will consider *I*^2^ values ≥50% and ≥75% indicative of substantial and serious heterogeneity, respectively.

#### 2.6.3 Data synthesis

We will provide a narrative synthesis of the results from the included studies, structured around the demographic characteristics of the participants, details of the experimental and control interventions, outcomes, and results. We will perform the meta-analysis using Review Manager software version 5.4 for Windows (Copenhagen, The Nordic Cochrane Center, the Cochrane Collaboration, 2020) if studies have used the same type of experimental intervention, comparison, and outcome measure. The data will be pooled using a random-effects model when the heterogeneity is significant (*I*^2^ value ≥50%), whereas a fixed-effects model will be used when the heterogeneity is non-significant or the number of studies included in the meta-analysis is very small, which would indicate that the estimate of between-study variance lacks precision.

#### 2.6.4 Subgroup analysis

If the necessary data are available, we will conduct a subgroup analysis according to the types of HM, duration of treatment, psychotropic drugs used, and patient age.

#### 2.6.5 Sensitivity analysis

If the results show high heterogeneity (*I*^2^ values ≥50%), we will conduct a sensitivity analysis.

#### 2.6.6 Publication bias

If there are >10 trials included in the meta-analysis, we will also assess evidence of publication bias using funnel plots.

#### 2.6.7 Summary of evidence

We will use the Grades of Recommendation, Assessment, Development, and Evaluation (GRADE) profiler version 3.6.1 (GRADE Working Group) to assess the quality of evidence.^[38]^ For the assessment scale, the confidence in each outcome will be divided into 4 levels: high, medium, low, and extremely low.

#### 2.6.8 Ethics and dissemination

As no individual data from patients will be involved in this study, it is exempt from ethical approval. The results of this study will be published in a peer-reviewed journal.

## 3 Discussion

PSI is a common complication in stroke survivors. It can not only affect recovery, QoL, and mortality but also increase the risk of related secondary diseases.^[16–18]^ As insomnia is a potentially modifiable risk factor, it is important to address PSI to improve stroke outcomes. However, few studies have investigated alternatives for the treatment of PSI, and current pharmacotherapy is associated with many side effects and little evidence is available regarding its efficacy. As HM exerts effects through multi-component, multi-target, and multi-pathway mechanisms, it has potential as an alternative hypnotic to conventional pharmacotherapy. Recent systematic reviews have shown that HM can significantly relieve insomnia.^[29–33]^ Therefore, we will perform an updated and expanded meta-analysis to provide reliable evidence regarding the efficacy and safety of HM in the treatment of PSI. This rigorous systematic review may provide optimal recommendations for the clinical treatment of PSI using HM.

Nonetheless, there may be some potential limitations in this study. First, as we will allow different types of HM prescription, heterogeneity regarding the intervention may be high. Second, as we include studies use not a placebo drug but conventional pharmacotherapy as a control, the quality of the RCTs may be low owing to performance bias.

## Author contributions

**Conceptualization:** Sang-Ho Kim.

**Data curation:** Sang-Ho Kim, Jung-Hwa Lim.

**Formal analysis:** Sang-Ho Kim.

**Investigation:** Sang-Ho Kim.

**Methodology:** Sang-Ho Kim.

**Project administration:** Sang-Ho Kim.

**Resources:** Sang-Ho Kim.

**Software:** Sang-Ho Kim.

**Supervision:** Jung-Hwa Lim.

**Validation:** Sang-Ho Kim.

**Writing – original draft:** Sang-Ho Kim.

**Writing – review & editing:** Jung-Hwa Lim.

## Supplementary Material

Supplemental Digital Content
