# Supplementary material for: Herbal medicine for post-stroke insomnia: A protocol for systematic review and meta-analysis
Source: Medicine (Baltimore). 2021 Jun 4;100(22):e26223. doi: 10.1097/MD.0000000000026223 (PMC8183778; doi:10.1097/MD.0000000000026223)
Supplement: Supplemental Digital Content [file medi-100-e26223-s001.docx]

**Appendix 1**. Search terms used in each database

**Medline via PubMed**

|  | Searches | Results |
| --- | --- | --- |
| #1 | “Sleep”[MH] OR “sleep wake disorders”[MH] OR sleep* OR insomnia* OR wakeful* OR sleepless* OR dyssomn* |  |
| #2 | "stroke"[MH] OR stroke |  |
| #3 | “Plants, Medicinal”[MH] OR “Drugs, Chinese Herbal”[MH] OR “Medicine, Chinese Traditional”[MH] OR “Medicine, Kampo”[MH] OR “Medicine, Korean Traditional”[MH] OR “Herbal Medicine”[MH] OR “Prescription Drugs”[MH] OR “traditional Korean medicine” OR “traditional Chinese medicine” OR “Traditional oriental medicine” OR “Kampo medicine” OR “alternative medicine” OR “complementary medicine” OR herb* OR decoction* OR botanic* |  |
| #4 | #1 AND #2 AND #3 |  |

**EMBASE via Elsevier**

|  | Searches | Results |
| --- | --- | --- |
| #1 | ‘Sleep’/exp OR ‘sleep’ OR ‘sleep disorder’/exp OR ‘sleep disorder’ OR ‘insomnia*’ OR ‘wakeful*’ OR ‘sleepless*’ OR ‘dyssomn*’ |  |
| #2 | 'cerebrovascular disease'/exp OR 'cerebrovascular disease' |  |
| #3 | 'stroke patient'/exp OR 'stroke patient' |  |
| #4 | ‘Stroke’ |  |
| #5 | ‘medicinal plant’/exp OR ‘medicinal plant’ OR ‘herbaceous agent’/exp OR ‘herbaceous agent’ OR ‘chinese medicine’/exp OR ‘chinese medicine’ OR ‘kampo medicine’/exp OR ‘kampo medicine’ OR ‘kampo medicine (drug)’/exp OR ‘kampo medicine (drug)’ OR ‘korean medicine’/exp OR ‘korean medicine’ OR ‘herbal medicine’/exp OR ‘herbal medicine’ OR ‘prescription drug’/exp OR ‘prescription drug’ OR ‘oriental medicine’/exp OR ‘oriental medicine’ OR ‘alternative medicine’/exp OR ‘alternative medicine’ OR ‘complementary medicine’ OR ‘herb’/exp OR ‘herb’ OR ‘decoction*’ OR ‘botanic*’ |  |
| #7 | #1 AND (#2 OR #3 OR #4) AND #5 |  |

**CENTRAL**

|  | Searches | Results |
| --- | --- | --- |
| #1 | MeSH descriptor: [Sleep] explode all trees |  |
| #2 | MeSH descriptor: [Sleep wake disorders] explode all trees |  |
| #3 | sleep* OR insomnia* OR wakeful* OR sleepless* OR dyssomn* |  |
| #4 | #1 OR #2 OR #3 |  |
| #5 | MeSH descriptor: [Stroke] explode all trees |  |
| #6 | (stroke):ti,ab,kw |  |
| #7 | #5 OR #6 |  |
| #8 | (#1 OR #2 OR #3) AND (#5 OR #6) |  |
| #9 | MeSH descriptor: [Plants, Medicinal] explode all trees |  |
| #10 | MeSH descriptor: [Drugs, Chinese Herbal] explode all trees |  |
| #11 | MeSH descriptor: [Medicine, Chinese Traditional] explode all trees |  |
| #12 | MeSH descriptor: [Medicine, Kampo] explode all trees |  |
| #13 | MeSH descriptor: [Medicine, Korean Traditional] explode all trees |  |
| #14 | MeSH descriptor: [Herbal Medicine] explode all trees |  |
| #15 | MeSH descriptor: [Prescription Drugs] explode all trees |  |
| #16 | traditional Korean medicine OR traditional Chinese medicine OR Traditional oriental medicine OR Kampo medicine OR alternative medicine OR complementary medicine OR herb* OR decoction* OR botanic* |  |
| #17 | #9 OR #10 OR #11 OR #12 OR #13 OR #14 OR #15 OR #16 |  |
| #18 | #8 AND #17 |  |

**AMED via EBSCO**

|  | Searches | Results |
| --- | --- | --- |
| #1 | Sleep[SU] OR sleep wake disorders[SU] OR sleep*[TX] OR insomnia*[TX] OR wakeful*[TX] OR sleepless*[TX] OR dyssomn*[TX] |  |
| #2 | stroke[SU] |  |
| #3 | stroke[TX] |  |
| #4 | Plants, Medicinal[SU] OR Drugs, Chinese Herbal[SU] OR Medicine, Chinese Traditional[SU] OR Medicine, Kampo[SU] OR Medicine, Korean Traditional[SU] OR Herbal Medicine[SU] OR Prescription Drugs[SU] |  |
| #5 | traditional Korean medicine[TX] OR traditional Chinese medicine[TX] OR Traditional oriental medicine[TX] OR Kampo medicine[TX] OR alternative medicine[TX] OR complementary medicine[TX] OR herb*[TX] OR decoction*[TX] OR botanic*[TX] |  |
| #6 | #1 AND ( #2 OR #3) AND (#4 OR #5) |  |

**CINAHL via EBSCO**

|  | Searches | Results |
| --- | --- | --- |
| #1 | Sleep[SU] OR sleep wake disorders[SU] OR sleep*[TX] OR insomnia*[TX] OR wakeful*[TX] OR sleepless*[TX] OR dyssomn*[TX] |  |
| #2 | stroke[SU] |  |
| #3 | stroke[TX] |  |
| #4 | Plants, Medicinal[SU] OR Drugs, Chinese Herbal[SU] OR Medicine, Chinese Traditional[SU] OR Medicine, Kampo[SU] OR Medicine, Korean Traditional[SU] OR Herbal Medicine[SU] OR Prescription Drugs[SU] |  |
| #5 | traditional Korean medicine[TX] OR traditional Chinese medicine[TX] OR Traditional oriental medicine[TX] OR Kampo medicine[TX] OR alternative medicine[TX] OR complementary medicine[TX] OR herb*[TX] OR decoction*[TX] OR botanic*[TX] |  |
| #6 | #1 AND ( #2 OR #3) AND (#4 OR #5) |  |

**PsycARTICLES via ProQuest**

|  | Searches | Results |
| --- | --- | --- |
| #1 | Exact("sleep wake disorders") |  |
| #2 | Exact("sleep") |  |
| #3 | SU(Sleep) OR SU(sleep wake disorders) OR 'sleep*' OR 'insomnia*' OR 'wakeful*' OR 'sleepless*' OR 'dyssomn*' |  |
| #4 | Exact("stroke") |  |
| #5 | Stroke |  |
| #6 | Exact("drugs, chinese herbal" OR "plants, medicinal" OR "prescription drugs" OR "medicine, chinese traditional" OR "alternative medicine" OR "medicinal herbs and plants") |  |
| #7 | 'traditional Korean medicine' OR 'traditional Chinese medicine' OR 'Traditional oriental medicine' OR 'Kampo medicine' OR 'alternative medicine' OR 'complementary medicine' OR 'herb*' OR 'decoction*' OR 'botanic*' |  |
| #7 | (#1 OR #2 OR #3) AND (#4 OR #5) AND (#6 OR #7) |  |

**OASIS**

|  | Searches | Results |
| --- | --- | --- |
| #1 | (뇌졸중 OR 중풍) AND (불면 OR 수면) AND 한약 |  |

**KISS**

|  | Searches | Results |
| --- | --- | --- |
| #1 | (뇌졸중 OR 중풍) AND (불면 OR 수면) AND 한약 |  |

**RISS**

|  | Searches | Results |
| --- | --- | --- |
| #1 | (뇌졸중 OR 중풍) AND (불면 OR 수면) AND 한약 |  |

**KMbase**

|  | Searches | Results |
| --- | --- | --- |
| #1 | (뇌졸중 OR 중풍) AND (불면 OR 수면) AND 한약 |  |

**KCI**

|  | Searches | Results |
| --- | --- | --- |
| #1 | (뇌졸중 OR 중풍) AND (불면 OR 수면) AND 한약 |  |

**CNKI**

|  | Searches | Results |
| --- | --- | --- |
| #1 | (SU='中风'+'脑卒中') AND (SU='失眠'+'不寐'+'不眠'+'不睡'+'不得眠'+'不得卧') AND (SU='中药'+'中医药'+'本草'+'汤'+'丸'+'散'+'方'+'颗粒'+'胶囊'+'自拟') |  |

**Wanfang data**

|  | Searches | Results |
| --- | --- | --- |
| #1 | (“中风” + “脑卒中”) * (“失眠” + “不寐” + “不眠” + “不睡” + “不得眠” + “不得卧”) * (“中药” + “中医药” + “本草” + “汤” + “丸” + “散” + “方” + “颗粒” + “胶囊” + “自拟”) |  |

**VIP**

|  | Searches | Results |
| --- | --- | --- |
| #1 | 题名或关键词=中风 或者 题名或关键词=脑卒中 并且 名或关键词=失眠 或者 题名或关键词=不寐 或者 题名或关键词=不眠 或者 题名或关键词=不睡 或者 题名或关键词=不得眠 或者 题名或关键词=不得卧 并且 题名或关键词=中药 或者 题名或关键词=中医药 或者 题名或关键词=本草 或者 题名或关键词=汤 或者 题名或关键词=丸 或者 题名或关键词=散 或者 题名或关键词=方 或者 题名或关键词=颗粒 或者 题名或关键词=胶囊 或者 题名或关键词=自拟 |  |

**CiNii**

|  | Searches | Results |
| --- | --- | --- |
| #1 | (stroke OR "stroke" OR 中風 OR 脳卒中) AND (Sleep OR insomnia* OR wakeful* OR sleepless* OR dyssomn* OR 不眠 OR [ふみん](https://ja.dict.naver.com/entry/jk/JK000000078287.nhn) OR [インソムニア](https://ja.dict.naver.com/entry/jk/LW46566.nhn) OR [ねぶそく](http://jpdic.naver.com/entry/user/a04ebd5b25e1146e152af0574cb94b73.nhn)) AND (“traditional Korean medicine” OR “traditional Chinese medicine” OR “Traditional oriental medicine” OR “Kampo medicine” OR “alternative medicine” OR “complementary medicine” OR herb* OR decoction* OR botanic* OR 漢方薬 OR ハーブ OR 散 OR 汤 OR 丸) |  |
